# Bi-directional association between sleep and outdoor active play among 10–13 year olds

**DOI:** 10.1186/s12889-018-5122-5

**Published:** 2018-02-07

**Authors:** Yingyi Lin, Michael M. Borghese, Ian Janssen

**Affiliations:** 1 Department of Public Health Sciences, Queen’s University, Kingston, ON K7L 3N6 Canada; 2 School of Kinesiology and Health Studies, Queen’s University, Kingston, ON K7L 3N6 Canada

**Keywords:** Child, Physical activity, Sleep

## Abstract

**Background:**

The purpose of this study was to investigate whether there is a bi-directional relationship between sleep characteristics (time in bed, sleep duration, sleep chronology, and sleep efficiency) and time spent participating in outdoor active play among children.

**Methods:**

Participants consisted of 433 children aged 10–13 years from Kingston, ON, Canada. Time in bed, sleep duration, sleep chronology, and sleep efficiency were measured for 8 consecutive nights using data from a sleep log and Actical accelerometer. Outdoor active play was measured for the 7 days that fell in between these 8 nights using a combination of data from accelerometers, global positioning system loggers, and geographic information systems. Generalized estimating equation models were used to assess the relationships between sleep characteristics and outdoor active play. These models accounted for the repeated measures nested within participants and adjusted for several confounders (e.g., age, sex, family income, neighborhood traffic and green space).

**Results:**

Time in bed, sleep duration, sleep chronology, and sleep efficiency were not significantly associated with the following day’s outdoor active play. There was a significant (*p* = 0.017) association between outdoor active play and the following night’s time in bed, which suggested that each hour increase in outdoor active play was associated with a 4.0 min increase in time in bed. Outdoor active play was not significantly associated with sleep duration, sleep chronology, or sleep efficiency.

**Conclusions:**

None of the sleep characteristics predicted the following day’s outdoor active play. Increase time spent in outdoor active play predicted a longer time in bed, but not sleep duration, sleep chronology, or sleep efficiency.

## Background

Insufficient time in bed, sleep duration, and sleep quality are associated with several health outcomes among children and adolescents including poor cognitive development, anxiety, depression and obesity [1–3]. Insufficient physical activity, particularly of a moderate-to-vigorous intensity, is also associated with a poor mental and physical health [4, 5]. Public health guidelines recommend that 5–13 year olds get 9–11 h of sleep per night, that 14–17 year olds get 8–10 h of sleep per night, and that 5–17 year olds get at least 60 min of moderate-to-vigorous physical activity per day [6–8]. It is concerning that a large proportion of children and adolescents do not get enough sleep or physical activity. In Canada, for instance, 20% of children and adolescents do not meet sleep recommendations [9], and only 9% meet physical activity recommendations on a daily basis [10]. Outdoor active play (OAP) is a type of physical activity that is particularly low among children living in industrialized countries [11, 12]. OAP refers to physical activity that is comprised of games or symbolic play that occurs outdoors and includes playground activities, ball games played in the street, and backyard games like tag and red rover. It is typically self-directed by children and involves little or no adult supervision.

Sleep and physical activity have historically been thought of as independent behaviors; however, there is a growing interest in determining whether they are connected. Research examining the possible connection between sleep and physical activity within school-aged children and adolescents has produced inconsistent findings. Studies have reported that nighttime sleep duration and quality positively [13], negatively [14, 15], or do not [16, 17] predict the next day’s physical activity. Similarly, studies have reported that physical activity during the day positively [15, 16, 18–20], negatively [15], or does not [16, 17, 19, 21] predict the subsequent night’s sleep duration and quality. These inconsistencies may in part reflect the lack of specificity of the physical activity variables being examined. That is, observational studies looking at the relationship between sleep and physical activity have captured the total time spent in all types of physical activity. Consideration should be given to specific types of physical activity because different types of physical activity may have different relationships with sleep, and these relationships could become obscured when total physical activity is considered [20]. For instance, because organized sports are often scheduled for the early morning and late evening, they might negatively impact sleep by disrupting when children can go to bed at night and when they need to wake up in the morning [22, 23]. However, if children do not have sufficient sleep the preceding night, their OAP the following day might be easily sacrificed. Conversely, OAP, an unscheduled activity, may benefit sleep because it is associated with factors associated with contributing to a peaceful sleep at night [24–26].

The purpose of this study was to investigate whether there is a bi-directional relationship between measures of sleep and OAP among 10- to 13-year-olds. Specifically, this study examined whether sleep characteristics (i.e., time in bed, sleep duration, sleep chronology, and sleep efficiency) at night was associated with the amount of OAP accumulated the following day, and whether OAP accumulated during the day was associated with sleep characteristics the following night.

## Methods

### Study participants

The study sample was from the Active Play Study. Data were collected between January 2015 and December 2016. To be included, participants must have been aged 10 to 13 years and lived and attended school in Kingston, Ontario, Canada. Children who did not speak English or French were excluded as were non-ambulatory individuals. Participants were recruited to ensure proportional representation of the city’s 10- to 13-year-old population by age, sex, and residence within its 12 electoral districts. An equal number of participants were studied in each of the four seasons. Recruitment strategies included word of mouth, social media, and advertisements posted and distributed in schools, stores, and community centers. Participants and a parent/guardian provided written informed consent prior to participation. The study was approved by the General Research Ethics Board at Queen’s University.

Twenty five (5.5%) of the 458 participants who participated in the Active Play Study were excluded from the present analyses because they did not have valid sleep and OAP measures for any of the 8 nights and 7 days (as explained below). Thus, the final sample consisted of 433 participants. Participants who were excluded from the analyses were similar in age (11.5 vs. 11.8 years) and ethnicity (90.5% vs. 88.0% white) to those who were included; however, there was a difference in gender distribution (68.0% vs. 49.2% male). Within these 433 participants, there were several days and nights with insufficient (invalid) data to determine sleep and/or OAP, and these nights and days were removed. The final number of observations were 2253 for the analyses where nighttime sleep characteristics predicted the following day’s OAP, and 2263 for the analyses where daytime OAP predicted the following night’s sleep characteristics.

### Data sources

Data sources included the following: 1) Participants wore an Actical accelerometer (Philips Respironics, Murrysville, PA) on their right hip for 7 days and 8 nights to continuously assess their movement in 15-s epochs; 2) During the same week, participants wore a Garmin Forerunner 220 Global Positioning System (GPS) watch (Garmin Ltd., Canton, Switzerland) which continuously recorded their longitude and latitude position; 3) During the same week, participants maintained a log wherein they recorded the times they went to bed at night and got up in the morning, the times they participated in organized sports and programs, the times they worked or did chores outdoors, and the times they removed their accelerometer or GPS watch; 4) Participants and one of their parents/guardians each completed a ~ 20-min long questionnaire on a tablet computer; 5) A series of physical measures (e.g., height, weight) were obtained; 6) Geographic information system (GIS) data for the city of Kingston were obtained from several sources; 7) Information on school calendars and schedules (e.g., start/end times, recess times) were obtained from school websites and by asking participants; 8) Sunrise/sunset times for the city of Kingston during each day of data collection were obtained from an online resource [27].

### Measurement of sleep

Four sleep characteristics were assessed to characterize the nocturnal sleep period: time in bed, sleep duration, sleep chronology, and sleep efficiency. They were measured over 8 consecutive nights. Time in bed (hours/day) was calculated as the difference between when participants turned off the lights to go to bed at night and when they got out of bed in morning, which they recorded on the log. These recorded times were verified, and corrected when necessary, by having a member of the research team visually inspecting the recorded log times against the Actical accelerometer data. In our laboratory, this process is highly reliable as 90% of the verified and corrected sleep times are within 10 min of each other based on repeated attempts completed by different researchers. Sleep chronology was determined using sleep midpoint, which was determined as the midpoint between the times participants turned off the lights to go to be at night and when they got out of bed in the morning.

Sleep duration (hours/day) was calculated as the total time spent sleeping during time in bed. To determine time spent sleeping, each of the 15-s long epochs (measurement intervals) from the Actical accelerometer was defined as either ‘sleeping’ or ‘awake’ based on a sleep likelihood score, and the time of the ‘sleeping’ epochs were summed. The sleep likelihood score was based on a weighted rolling average of the count value for the epoch in question and the 8 epochs that proceed and follow it. Sleep efficiency (%) was calculated as the ratio of sleep duration to time in bed [28, 29]. The method of estimating sleep/awake status using a waist-worn Actical accelerometer was developed and cross-validated in a sub-study of 50 children from the present study. Results from the sub-study showed that there is no significant difference in sleep efficiency estimates between the waist-word Actical accelerometer and a wrist worn accelerometer developed specifically to assess sleep characteristics (89.0% vs 88.7%, *p* = 0.665] [30].

### Measurement of OAP

The method for assessing OAP was developed by our laboratory and was one of the main objectives of the Active Play Study [Borghese MM, Janssen I. Development of a measurement approach to assess time children participate in outdoor active play, organized sport, active travel, and curriculum-based physical activity, submitted]. This method used data from several sources and involved a combination of automated steps, manual checks and corrections.

First, times recorded in the log (start and end times for sleep, organized sports, outdoor chores, and accelerometer non-wear periods) were checked by visually inspecting these times within the accelerometer data using Actical 3.10 software (Philips Respironics, Murrysville, PA). If necessary, corrections were made to the recorded times.

Second, Personal Activity and Location Measurement (PALMS) software (Center for Wellness and Population Health Systems, University of California, San Diego, CA) was used to merge the accelerometer and GPS data based on each 15-s accelerometer epoch. PALMS identified periods of time with missing GPS latitude and longitude coordinates, which occurred if the satellite signal was lost (e.g., when a participant entered a large building). When possible, missing geospatial coordinates were imputed by the research team by using Google Maps (Google, Mountain View, CA) and street view images to determine where the participant was immediately before the signal was lost and immediately after the signal returned. For instance, if the signal was lost for 30 min when a participant was in a building, the latitude and longitude coordinates for the center of that building were imputed for all 15-s epochs that occurred during that 30 min.

Third, PALMS software used a validated algorithm to identify all vehicle and non-vehicle (e.g., walking, bicycling) trips [31], and all 15-s epochs that occurred during trips were flagged. Trip detection was based on distance traveled over time (i.e., travel ≥100 m over ≥180 s at a speed of ≥1 km/h). Trip modality was determined by the 90th percentile of travel speed (walking = 1 to 9.99 km/h, cycling = 10 to 24.99 km/h, vehicle = ≥ 25 km/h). Each of these trips identified by PALMS were checked by visually inspecting all of the GPS coordinates for that trip in Google Maps. During these visual inspections we identified and then deleted a number of false positive trips. An example of a false positive trip is a trip identified by PALMS that, upon visual inspection, was found to occur on school grounds during the recess period. This example reflected OAP and not active transportation. During our visual inspections of the GPS data we did not encounter false negative trips, and therefore we did not have a need to identify trips that were not captured by the PALMS algorithm.

The data from each participant was then exported from PALMS into ArcMap version 10.4 software (Esri, Redlands, CA). The longitude and latitude coordinates for each epoch were geocoded and a map layer of building footprints for the city of Kingston was used to determine whether each 15-s epoch was indoors (in a building) or outdoors (not in a building). The files for all participants were then merged together using SAS version 9.4 statistical software (SAS Inc., Carry, NC).

The merged file contained over 19 million rows, with > 40,000 rows per participant (i.e., one row of data for each 15-s long epoch that occurred over the 7 day/8 night measurement period). Additional “time” information was merged into the master file, including 1) the sleep, organized sport, and chore/work times in the logs; 2) the start and end times of the school day and school recess times; and 3) whether each day represented a school day or a non-school day (weekend or holiday).

A SAS program was then developed to determine the number of minutes spent in OAP on each measurement day. The SAS program started by identifying and deleting all 15-s epochs for the days in which there was insufficient (< 10 h) accelerometer and GPS watch wear time during waking hours [32, 33]. The program then flagged all 15-s epochs that could not have occurred during OAP because it occurred during one or more of the following conditions: 1) time in bed, 2) indoors, 3) school curriculum time (but not recess time) on a school day, 4) a vehicle trip or non-vehicle trip, 6) while participating in an organized sport, or 7) while performing work or chores. All of the 15-s epochs that were not flagged in the proceeding step were then classified as either occurring during OAP or as sedentary time spent outdoors using a specifically designed algorithm that has a specificity of 85%, sensitivity of 85%, and positive predictive value of 99% for identifying OAP. This algorithm is not based on a single epoch intensity cut-off since OAP includes sedentary, light, and moderate-to-vigorous intensity movements. Rather, the algorithm is a function of the count value for the epoch in question, centered and forward rolling averages for the surrounding epoch count values, the duration of outdoor time session the epoch was contained within, and interactions of these variables. After the OAP epochs were identified, they were summed to determine the daily total. These daily OAP values were exported into a dataset with multiple observations for each participant (e.g., one row per day).

### Confounders

Several factors that are associated with both sleep and physical activity and which may confound the bi-directional relationship between these behaviors were considered, including characteristics of the participant, their family, their neighborhood environment, and characteristics that reflected the calendar day when the sleep and physical activity measures were obtained. Participant factors consisted of biological sex (male or female); age (continuous); ethnicity (white or non-white including mixed race); the presence of a chronic medical condition (yes or no); body mass index (BMI) z-score based on the World Health Organization growth reference (continuous) [34]; and biological maturity using the maturity offset method (continuous) [35]. Family factors consisted of annual family income (≤ $50,000, $50,001–100,000, > $100,000, or not reported) and the number of parents in the household (single or dual parent). Neighborhood environment factors included the proportion of land area within a 1 km distance of the home devoted to green space (continuous) and a traffic volume index, which was calculated as: [(km of arterial roads * average daily vehicle counts on arterial roads in Kingston) + (km of collector roads * average daily vehicle counts on collector roads in Kingston) + (km of local roads * average daily vehicle counts on local roads in Kingston)] / total road distance in buffer in km (continuous). Finally, characteristics of the day the sleep and physical activity measures were obtained including the type of day (school day, a non-school day in which the participant was enrolled in an organized day camp program, or other non-school days such as weekends and holidays), minutes/day of daylight hours, minutes/day spent participating in the other domains of physical activity (i.e., organized sports, active transportation, physical activity during school curriculum); and minutes/day of accelerometer and GPS watch wear time during waking hours.

### Preparation of datasets

Two data sets were formed for the statistical analyses. Each dataset had up to 7 repeated sleep - OAP or OAP - sleep pairings for each participant. While the sleep and OAP characteristics for each participant differed for each row, because their sleep and OAP varied from day to day, the covariate data were repeated for all 7 rows for each participant within each dataset, with the exception of the daylight hours, type of day, and other physical activity domain variables, which varied for each row.

### Statistical analysis

All statitical analyses were completed using SAS version 9.4 software. A *p*-value of < 0.05 was used to denote statistical significance. Standard descriptive statistics were used to describe the sample. Generalized estimating equation (GEE) models with a first order autoregressive matrix [AR(1)] were performed to assess the relationship of interest, which accounted for the repeated measures nested within participants and were adjusted for confounders. In analyses testing whether nighttime sleep was associated with OAP the following day, sleep characteristics represented the within-person independent variables and OAP the within-person dependent variable. Since ~ 20% of the days had zero values for OAP, a negative binomial distribution and a logit link were specified to adjust for the over-dispersion of non-zero values, and non-zero values were rounded to integer values to mimic count data. In analyses testing whether OAP during the day was associated with sleep characteristics the following night, OAP represented the within-person independent variable and sleep characteristics the within-person dependent variables. Three models were fit for each relationship: an unadjusted model, a partially adjusted model (adjusted for participant characteristics only), and a fully adjusted model (adjusted for participant characteristics, family characteristics, neighborhood environment, and characteristics of the measurement day).

## Results

Demographic information of the 433 participants are in Table 1 and the sleep and physical activity characteristics are in Table 2. There was an even split of 10, 11, 12, and 13 year olds and of boys and girls. The majority (90.5%) were white. The median (25th, 75th percentile) time in bed and sleep duration were 9:31 (9:02, 9:58) and 8:29 (7:59, 8:59) hours:minutes, respectively. The median sleep midpoint (chronology) was 2:44 (2:14, 3:29) AM. The median sleep efficiency was 90.0% (86.3, 92.5%). The median OAP was 32.7 (15.8, 54.2) minutes per day and for 20% of the days no OAP occurred.

**Table 1:** Demographic characteristics of study participants (*n* = 433)

Variable	N	%
Age (years)		
10	112	25.9
11	108	24.9
12	110	25.4
13	103	23.8
Sex		
Male	213	49.2
Female	220	50.8
Race		
White	392	90.5
Non-white	41	9.5
Body mass index status		
Underweight	8	1.9
Normal	307	70.9
Overweight	73	16.9
Obese	45	10.4
Chronic condition		
No	263	60.7
Yes	170	39.3
Family structure		
Single parent	59	13.6
Dual parent	371	85.7
Not reported	3	0.7
Family income ($ Canadian per year)		
≤ 50,000	67	15.5
50,001–100,000	118	27.3
> 100,000	196	45.3
Not reported	52	12.0

**Table 2:** Sleep and physical activity characteristics of study participants (*n* = 433)

Variable	Median (25th, 75th percentile)
Sleep	
Time in bed (hours:minutes)	9:31 (9:02, 9:58)
Sleep duration (hours:minutes)	8:29 (7:59, 8:59)
Sleep midpoint (hours:minutes)	2:44 (2:14, 3:29)
Sleep efficiency (%)	90.0 (86.3, 92.5)
Physical activity (minutes/day)	
Outdoor active play (minutes/day)	32.7 (15.8, 54.2)
Organized sports (minutes/day)	18.8 (0.0, 48.6)
Curriculum-based physical activity (minutes/day)	9.8 (2.1, 16.6)
Active transportation (minutes/day)	12.2 (5.4, 21.3)

The associations between nighttime sleep and the following day’s OAP are shown in Table 3. The β coefficients in the table represent the % change in OAP for every one hour increase in time in bed, sleep duration, and sleep chronology and every 1% increase in sleep efficiency. After adjusting for relevant covariates, none of the four nighttime sleep characteristics (time in bed, sleep duration, sleep chronology, sleep efficiency) were significantly associated with the following day’s OAP. In a sensitivity analysis that was limited to children who did not meet sleep duration recommendations (i.e., average nightly time in bed < 9 h), similar results were observed for all four sleep characteristics (*p* values for fully adjusted models were 0.066 for time in bed, 0.329 for sleep duration, 0.890 for sleep chronology, and 0.131 for sleep efficiency). The associations between daytime OAP and the following night’s sleep are shown in Table 4. The β coefficients in the table represent the change in sleep characteristics (hours for time in bed, sleep duration, and sleep chronology and % for sleep efficiency) for every 1 min increase in OAP. After adjusting for covariates, OAP during the day was significantly (*p* = 0.017) associated with time in bed on the following night. Extrapolation of the β coefficients indicated that each 60 min/day increase in OAP was associated with an additional 4 min/night increase in time in bed. The associations between OAP and sleep duration approached but did not reach statistical significance, and OAP was not associated with sleep efficiency or sleep chronology. In a sensitivity analysis that was limited to days when children accrued zero minutes of OAP there were no significant associations between OAP and any of the four sleep characteristics (*p* values for fully adjusted models were 0.632 for time in bed, 0.850 for sleep duration, 0.546 for sleep chronology, and 0.632 for sleep efficiency).

**Table 3:** Associations between nighttime sleep characteristics and OAP the following day (*n* = 432 participants and 2253 sleep - OAP pairings)

Sleep Characteristic	β (95% confidence interval)	*P* value
Time in bed		
Model 1^a^	0.027 (− 0.025, 0.079)	0.311
Model 2^b^	− 0.008 (− 0.062, 0.046)	0.779
Model 3^c^	0.009 (− 0.046, 0.063)	0.760
Sleep duration		
Model 1^a^	0.017 (− 0.033, 0.067)	0.515
Model 2^b^	− 0.023 (− 0.079, 0.032)	0.413
Model 3^c^	− 0.009 (− 0.064, 0.046)	0.745
Sleep chronology		
Model 1^a^	− 0.053 (− 0.122, 0.016)	0.136
Model 2^b^	− 0.008 (− 0.073, 0.058)	0.820
Model 3^c^	− 0.060 (− 0.136, 0.016)	0.123
Sleep efficiency		
Model 1^a^	− 0.004 (− 0.013, 0.005)	0.405
Model 2^b^	− 0.008 (− 0.018, 0.002)	0.119
Model 3^c^	− 0.008 (− 0.017, 0.002)	0.104

^a^Unadjusted model. Not adjusted for covariates

^b^Partially adjusted model. Adjusted for participant characteristics only (sex, age, race, maturity offset, chronic medical conditions, body mass index)

^c^Fully adjusted model. Adjusted for participant characteristics (sex, age, race, maturity, chronic medical conditions, body mass index), family characteristics (family income, number of parents in household), neighborhood environment characteristics (green space, traffic volume), and characteristics of the day sleep and outdoor active play were measured (weekday/weekend/holiday, daylight hours, time spent in other types of physical activity, accelerometer and GPS watch wear time)

**Table 4:** Associations between OAP during the day and the following night’s sleep characteristics (*n* = 433 participants and 2263 OAP - sleep pairings)

Sleep Characteristic	Β (95% confidence interval)	*P* value
Time in bed		
Model 1^a^	0.002 (0.001, 0.003)	0.006
Model 2^b^	0.001 (− 0.000, 0.002)	0.059
Model 3^c^	0.002 (0.000, 0.003)	0.017
Sleep duration		
Model 1^a^	0.001 (0.000, 0.003)	0.016
Model 2^b^	0.001 (− 0.000, 0.002)	0.151
Model 3^c^	0.001 (− 0.000, 0.002)	0.057
Sleep chronology		
Model 1^a^	0.000 (− 0.001, 0.001)	0.893
Model 2^b^	0.000 (− 0.001, 0.001)	0.387
Model 3^c^	− 0.000 (− 0.001, 0.001)	0.618
Sleep efficiency		
Model 1^a^	0.001 (− 0.004, 0.006)	0.750
Model 2^b^	− 0.000 (− 0.005, 0.005)	0.980
Model 3^c^	− 0.000 (− 0.005, 0.004)	0.868

^a^Unadjusted model. Not adjusted for covariates

^b^Partially adjusted model. Adjusted for participant characteristics only (sex, age, race, maturity offset, chronic medical conditions, body mass index)

^c^Fully adjusted model. Adjusted for participant characteristics (sex, age, race, maturity, chronic medical conditions, body mass index), family characteristics (family income, number of parents in household), neighborhood environment characteristics (green space, traffic volume), and characteristics of the day sleep and outdoor active play were measured (weekday/weekend/holiday, daylight hours, time spent in other types of physical activity, accelerometer and GPS watch wear time)

## Discussion

To our knowledge, this is the first study to examine the associations between sleep and OAP in children. We used objective tools and detailed logs to measure these behaviors. The statistical analyses allowed us to examine the temporal and bi-directional associations between sleep and OAP. Our results indicate that the sleep characteristics did not predict OAP. OAP was a modest predictor of time in bed but it was not associated with sleep duration, sleep chronology, or sleep efficiency.

Our finding that time in bed, sleep duration, sleep chronology, and sleep efficiency were not positively associated with the following day’s OAP is consistent with other observational studies that measured total physical activity or physical activity of a moderate-to-vigorous intensity [14–16, 36]. Conversely, a randomized crossover trial reported that children were about 4% more active in the days following a 1.5 h increase in time in bed versus the days following a 1.5 h decrease in time in bed [13]. A possible explanation as to why the experimental study found associations that were not observed in observational studies is that the 3 h difference in time in bed in the experimental study is a drastic change that does not reflect the normal night-to-night variability in sleep. As evidence of this, in our study the night-to-night difference in time in bed only reached 3 h for 3% of the observations. It is also worth noting that in the experimental study the 1.5 h difference in time in bed between the baseline and sleep increased (or decreased) conditions did not lead to a change in physical activity [13].

The association between daytime OAP and time in bed was statistically significant; however, the strength of the association was not clinically significant. In fact, an hour/day increase in OAP, a volume of OAP that was almost double the average of our sample, was associated with only a 4 min/night increase in time in bed without a corresponding change in sleep duration or efficiency. Thus, our findings suggest that it is unlikely that OAP interventions would have a meaningful impact on sleep, unless these interventions were able to change OAP by several hours/day, which is doubtful. It is important to note that the duration and quality of sleep in the children in this sample were very good, and it is possible that there was a ceiling effect and that sleep behaviors could not be further improved by engaging in more outdoor active play. Five other studies have investigated the acute effects of daytime physical activity on sleep characteristics in children using objective measurements [15, 16, 18, 19, 36]. The only one of these studies that found a meaningful association was Dworak et al.’s experimental study of 11–13 year olds [18]. The physical activity in that experimental study consisted of performing 30 min of continuous vigorous intensity exercise on a cycle ergometer in a laboratory. While effective at improving sleep, that pattern (continuous) and intensity (vigorous only) of physical activity is not typically observed in children [37], and it does not approximate the sporadic movement pattern and intensity of OAP. In fact, evidence suggests that only 27% of the time children participate in OAP is spent at a moderate-to-vigorous intensity [38].^57^

Our study was not devoid of limitations. First, the generalizability of the results may be limited to 10- to 13-year-olds without a diagnosed sleep disorder. Second, although accelerometers provide valid estimates of sleep duration and efficiency [39], our study’s internal validity may be compromised because sleep variables were not assessed via polysomnography, the gold standard. With that being said, accelerometers can be used in free-living conditions, while polysomnography measures need to be obtained in laboratory or clinical settings and these settings and the equipment could disrupt normal sleep behaviors. It is also important to mention that our sleep assessments only included the nighttime sleep session and did not include any naps that some of the children may have had. Also, it is possible that OAP was misclassified due to the limitations of accelerometer and GPS device. For example, if a participant removed their accelerometer while engaging in water-based OAP, their OAP would have been underestimated. Since the measurement bias of both sleep and OAP were likely non-differential, the associations of interest were biased towards the null. Furthermore, our OAP measure considered the total duration of all OAP sessions and we did not specifically examine OAP bouts of a set duration. Finally, although we investigated the relationships temporally using the appropriate exposure time window, temporality on its own does not equate to causality. More intervention research is needed to learn more about the causal nature of the relationships between sleep and physical activity in children.

## Conclusion

This study assessed the temporal and bi-directional relationships between sleep characteristics and OAP in a sample of 10- to 13-year-olds. Daytime OAP was weakly associated with time in bed on the following night. None of the other associations were statistically or clinically significant.

## Abbreviations

GEE: Generalized estimating equation
GIS: Geographic information systems
GPS: Global position system
OAP: Outdoor active play
